# Transcriptomic Analysis of Intestinal Tissues from Two 90-Day Feeding Studies in Rats Using Genetically Modified MON810 Maize Varieties

**DOI:** 10.3389/fgene.2017.00222

**Published:** 2017-12-19

**Authors:** Jutta Sharbati, Marc Bohmer, Nils Bohmer, Andreas Keller, Christina Backes, Andre Franke, Pablo Steinberg, Dagmar Zeljenková, Ralf Einspanier

**Affiliations:** ^1^Institute of Veterinary Biochemistry, Freie Universität Berlin, Berlin, Germany; ^2^Chair for Clinical Bioinformatics, Saarland University, Saarbrücken, Germany; ^3^Institute of Clinical Molecular Biology, Christian-Albrechts-Universität zu Kiel, Kiel, Germany; ^4^Institute for Food Toxicology and Analytical Chemistry, University of Veterinary Medicine Hannover, Hannover, Germany; ^5^Faculty of Public Health, Slovak Medical University in Bratislava, Bratislava, Slovakia

**Keywords:** transcriptomics profiles, pathway-analysis, rat feeding-trial, GM-plant, MON810-maize

## Abstract

**Background:** Global as well as specific expression profiles of selected rat tissues were characterized to assess the safety of genetically modified (GM) maize MON810 containing the insecticidal protein Cry1Ab. Gene expression was evaluated by use of Next Generation Sequencing (NGS) as well as RT-qPCR within rat intestinal tissues based on mandatory 90-day rodent feeding studies. In parallel to two 90-day feeding studies, the transcriptional response of rat tissues was assessed as another endpoint to enhance the mechanistic interpretation of GM feeding studies and/or to facilitate the generation of a targeted hypothesis. Rats received diets containing 33% GM maize (MON810) or near-isogenic control maize. As a site of massive exposure to ingested feed the transcriptomic response of ileal and colonic tissue was profiled via RT-qPCR arrays targeting apoptosis, DNA-damage/repair, unfolded protein response (UPR). For global RNA profiling of rat ileal tissue, we applied NGS.

**Results:** No biological response to the GM-diet was observed in male and in female rat tissues. Transcriptome wide analysis of gene expression by RNA-seq confirmed these findings. Nevertheless, gene ontology (GO) analysis clearly associated a set of distinctly regulated transcripts with circadian rhythms. We confirmed differential expression of circadian clock genes using RT-qPCR and immunoassays for selected factors, thereby indicating physiological effects caused by the time point of sampling.

**Conclusion:** Prediction of potential unintended effects of GM-food/feed by transcriptome based profiling of intestinal tissue presents a novel approach to complement classical toxicological testing procedures. Including the detection of alterations in signaling pathways in toxicity testing procedures may enhance the confidence in outcomes of toxicological trials. In this study, no significant GM-related changes in intestinal expression profiles were found in rats fed GM-maize MON810. Relevant alterations of selected cellular pathways (apoptosis, DNA damage and repair, UPR) pointing toward intestinal toxicity of the diets were not observed. Transcriptomic profiles did not reveal perturbations of pathways associated with toxicity, underlining the study results revealed by classical OECD endpoints.

## Introduction

Controversial public discussions have been raised immediately after introducing genetically modified (GM) plants on the market some decades ago and are still under debate (Fagerstrom et al., 2012). Hence numerous scientific approaches were suggested to investigate possible health impacts of commercialized GM food/feed components providing a deeper knowledge on their uptake, disposition and effects (Nicolia et al., 2014). Distinct national and international regulations prompted specific risk assessment procedures before market placement of GM products, like compositional analysis and/or animal feeding studies. Within the European Union, 90-day feeding studies with whole food/feed performed with rodents are mandatory (EC Regulation No. 503/2013) and their study design is generally based on OECD test guidelines and EFSA recommendations (OECD TG 408, EFSA 2011).

MON810 is a well established example for a commercialized GM maize event expressing the truncated Cry1Ab insecticidal protein derived from the Bacillus thuringiensis (Bt) toxin. Bt-toxin containing crops were developed for pest control targeting the European Corn Borer and represent one of the most widely grown transgenic maize varieties. Extensive acute and chronic toxicology studies investigating such Bt-containing plants have not found increased health risks for human or animals (Betz et al., 2000).

Previous studies demonstrated the presence of Cry1Ab protein throughout the intestinal tract of pigs (Walsh et al., 2011) and calves (Chowdhury et al., 2003), while Cry1Ab protein quantification revealed the highest concentration in colonic digesta in pigs. In addition, while gastric fluids digest the Cry1Ab toxin, it is resistant to intestinal fluids (Guimaraes et al., 2010). In contrast, Cry1Ab protein was not detected in other organs (Walsh et al., 2011).

The intestinal tract in total and the small intestine in particular, have a very large surface area, which makes the latter a major site of exposure to orally ingested substances or mixtures. The intestinal tract expresses a wide variety of metabolic enzymes and is assumed to be the most important site of extra-hepatic metabolism of xenobiotics (Thelen and Dressman, 2009). Notably, the gut plays an important role in the complex mechanisms of immune system homeostasis. It represents the largest mass of lymphoid tissue in the body, and the gut associated lymphoid tissue (GALT) plays a key role in hypersensitivity reactions to food proteins (Vighi et al., 2008). In particular the ileum is characterized by the presence of Peyer’s patches, while the colon plays an important role in disease development, e.g., colon cancer.

The present study was part of the project “GMO Risk Assessment and Communication of Evidence (GRACE)” funded by the European Commission within the 7th Framework Program. In this study, we analyzed intestinal tissue from two independent 90-day feeding trials using two commercial varieties of maize MON810 with the same genetic events, but different genetic backgrounds (Zeljenkova et al., 2014). The first trial (study A) studied Monsanto MON810 maize, the second trial (study B) included Pioneer MON810 maize. We aimed to evaluate if gene expression analysis of rat intestinal tissues may facilitate hypothesis generation and/or provides an added value for safety assessment of GM-food/feed. For this aim, we applied targeted analyses of gene expression signatures potentially associated with intestinal disease and toxicity. In addition, we applied next generation sequencing analysis (RNAseq) to rat ileal tissues to generate data sets reflecting potential perturbations of global intestinal signaling pathways by GM-food/feed. This untargeted approach may enable the discovery of unintended cellular effects by identifying molecular patterns associated with known pathways of disease or toxicity. This work represents a novel approach for predicting intestinal toxicity based on in depth analysis of RNA-based differential expression patterns.

## Materials and Methods

### Rat Feeding Trials

Diet preparation and study design of the rat feeding trials were described in a previous publication (Zeljenkova et al., 2014) following OECD test guideline 408. Briefly, intestinal tissue was collected after two repeated-dose 90-day oral toxicity studies in rats (referred to as studies A and B). The animal trials were performed in compliance with GLP in the experimental animal house at the Department of Toxicology of the Slovak Medical University in Bratislava and approved by the ethics commitee of the Veterinary State Administration, Slovak Republic Ro-4372/12-221. The two studies used maize materials with the MON810 event but with different varieties having different genetic backgrounds (study A = MON810 from Monsanto; study B = MON810 from Pioneer). For RNA analysis, we used intestinal tissue (ileum, ascending colon) from high dose study groups, which were fed diets containing 33% GM or 33% near-isogenic control maize. For study A, male and female animals were sampled on four consecutive days, each day starting with animals from the control group and finishing with animals from the GM-maize fed group. Sampling was performed over a period of 10 h. A similar sampling scheme was applied in study B, but the interval between sacrifice of the first and last animal was 8 h. Due to the high number of animals per group a variation in time of sacrifice was given as a total of 160 rats were introduced in a randomized block design.

### RNA Extraction and mRNA Quantification by RT-qPCR

Tissue samples from 10 animals per group and sex were used (*n* = 10). In total, 20 male and 20 female rats were examined, one half being fed the 33% GM maize feed and one half being fed the 33% isogenic maize feed. The tissue samples (ileum, colon) were dissected after sacrifice, immediately frozen in liquid nitrogen and stored at -80°C. Total RNA from total ileum and colon macro-dissections (∼250 mg wet-weight) was isolated using the mirVana^TM^ miRNA Isolation Kit (Ambion), according to the manufacturer’s protocol. The RNA quantity was determined with the Nanodrop 1000 Spectrophotometer (Thermo) whereas the quality was controlled as previously described via the 2100 Bioanalyzer Instrument (Agilent Technologies) (Sharbati et al., 2010). Quantification of mRNA expression was performed by RT-qPCR as described earlier (Sharbati et al., 2011), with some modifications. One microgram of total RNA was reverse-transcribed using the RevertAid^TM^ M-MuLV Reverse Transcriptase (Fermentas GmbH) in 20 μl total volume using random hexamers. SYBR Green qPCR was performed using the SensiMix DNA Kit (Quantace Ltd.) and 0.2 μM of gene specific primers, synthesized by Sigm–Aldrich (electronic Supplementary Table S1). The first step of amplification was denaturation at 95°C for 2 min, followed by 40 cycles for 15 s at 95°C, for 10 s at 60°C and for 10 s at 72°C. Melting curve analysis allowed testing for specificity of each qRT-PCR. The PCR reactions were optimized for each primer pair by the use of serial diluted PCR products for standard curves with a dynamic range between 5 and 8 orders of magnitude and 80–110% efficiency at 60°C annealing temperature. All targeted genes were verified by sequencing of purified amplicons. All transcripts were quantified by triplicate measurements of 1 μl 1:5 diluted cDNA in 10 μl final reaction volume using either the StepOnePlus^TM^ Real-Time PCR System (Life Technologies) or the PikoReal^TM^ Real-Time PCR System (Thermo Scientific). Normalization of expression data was performed using three stably expressed reference genes. The RefFinder algorithm (Xie et al., 2012) was used to determine the most useful reference genes of the gene set: *ACTB, B2M, HPRT1, LDHA1, and RPLP1*. The relative gene expression was obtained by calculating the ΔΔ*C*q values between the control and treatment animals.

### RNA Sequencing

For RNA sequencing eight animals per diet and sex were used (*n* = 8). In total 16 females and 16 males were included, one half being fed the 33% GM maize feed and one half being fed the 33% isogenic maize feed. TruSeq libraries were prepared using the Illumina “TruSeq Stranded Total RNA with Ribo-Zero Gold” library kit from 500 ng of intact whole RNA samples with RIN (RNA Integrity Number) values of at least 5.6 and OD260/280 of at least 1.8. All RNA samples from both male and female biological replicates were prepared and subsequently analyzed using the Agilent 2200 Tapestation. Ribosomal depletion was performed using library preparation kit. Sequencing was carried out on Illumina HiSeq 2500 instruments as a 2 × 125 bp run. RNAseq mass data have been submitted to the NIH resource via SRA submission^1^ under the ID SUB2976951 Bioproject PRJNA400118 (accession numbers SAMN07556813 – SAMN07556876).

### Immunofluorescence Staining

In the case of paraffin embedded tissues a standard rehydration protocol for 6 μm sections was applied as described earlier (Scholven et al., 2009). To reduce auto-fluorescence, sections were immersed in 0.1% Sudan Black diluted in 70% ethanol for 20 min at RT, followed by washing 5 min in 50% ethanol and rehydration in PBS (PBS including 1% BSA) 10 min at RT. Antigen retrieval was achieved by boiling in 10 mM citrate buffer, pH 6, for 40 min. Non-specific binding of the primary antibody was blocked by incubation in 10% goat serum in PBS (1% BSA in PBS) for 1 h at RT. Mucous and cell membranes were stained using an Alexa Fluor 594 conjugate of wheat germ agglutinin (Life Technologies) at a concentration of 5.0 μg/mL HBSS for 15 min at RT, followed by washing for 20 min in PBS. Rabbit monoclonal anti-NR1D1 antibody (Abcam, EPR10376, 0.086 mg/ml) was used at a 1:50 dilution (PBS with 1% BSA). A goat anti-rabbit IgG conjugated to Alexa Fluor 488 (pre-absorbed, Abcam) was used as the secondary antibody at a dilution of 1:400 for 1 h at RT. Incubations without the primary antibody, or with rabbit IgG1, were performed as negative controls. Nuclei were counterstained using 200 ng/ml DAPI (Roche) for 10 min, and sections were embedded in 50% glycerol in PBS.

### Western Blot

Consistent with the RNA analysis, we analyzed protein samples of eight rats (same individuals) per study group and sex of study A (except for only seven males of the control group, due to limited tissue material). Intestinal tissues were homogenized in RIPA buffer (Cell Signaling Technology) supplemented with 1 mM PMSF and 1% (v/v) protease inhibitor cocktail (Calbiochem). Protein concentration was determined using a 2-D Quant Kit (GE Healthcare). Twenty micrograms of total protein were separated on a 12% (w/v) polyacrylamide gel under reducing conditions. Subsequently, proteins were transferred onto a nitrocellulose blotting membrane 0.45 μm (Sartorius) by semi-dry electroblotting for 70 min at 1.2 mA/cm^2^. The membrane was blocked for 1h with 5% skim milk (Carl Roth) in TBST. Overnight incubation was performed at 4°C with a monoclonal anti-rabbit NR1D1 antibody (Abcam, EPR10376, 0.086 mg/ml) at a 1:1000 dilution or a HPRT monoclonal anti-rabbit antibody (Abcam) at a 1:7500 dilution in TBST 3% BSA. Donkey anti-rabbit IgG horseradish peroxidase-conjugated secondary antibody (Amersham Biosciences) was used at a dilution of 1:20000 for 2 h. Immunoreactive proteins were visualized using enhanced chemiluminescence (ECL Select, Amersham), and the obtained bands were visualized using the documentation system Fusion SL (Vilber Lourmat). Signals were quantified using the program Bio1D (Vilber Lourmat). For statistical analysis, the means (triplicate individual measurements) of treated samples and non-treated controls were calculated and were subjected to the Mann–Whitney-*U*-Test, a *p* < 0.05 being required for significance.

### Statistical and Bioinformatical Methods

For RT-qPCR statistical analysis, the log two ratio of means (triplicate RT-qPCR measurements) of treated samples and non-treated controls were calculated and were subjected to the Kruskal–Wallis-test followed by the Mann–Whitney *U*-test as *post hoc* test, *p* < 0.05 being required for significance using IBM SPSS Statistics 20. Volcano plots as well as the comparison of NGS and RT-qPCR data (Spearman’s rank correlation) were performed by using Microsoft Excel 2010. To evaluate the RNAseq data, we applied Tophat (version 2.0) and Cufflinks (version 2.2) as described (Trapnell et al., 2012) with default parameters. Cufflinks also contains the programs Cuffdiff and CummeRbund to compute and visualize the differential expression between the groups. Cuffdiff computes variance estimates given multiple replicates and uses them to calculate the significance of the observed expression differences between the groups. Besides the raw computed *p*-value, Cuffdiff also outputs *p*-values corrected for multiple testing (FDR method (Benjamini and Hochberg, 1995)) and log2 fold changes, which we used to extract the differentially expressed genes for downstream analyses. Gene Ontology (GO) biological process analysis of genes with highest fold changes was performed using DAVID (Huang da et al., 2009). As the threshold for gene enrichment we applied an EASE Score (modified Fisher Exact *P*-Value) of 0.1. We ranked genes according to modified fisher exact *p*-values (the smaller the more enriched). In **Figures 2–4** we showed 10 genes with lowest modified fisher exact *p*-value. To explore the biological context of differentially expressed genes, we performed the GO classification, making use of the category BP_Fat (biological process). Network analysis was applied to predict protein-protein interaction. We imported genes with highest fold changes to STRING database and visualized the networks by Cytoscape software (Shannon et al., 2003).

## Results

### Targeted Analysis of Gene Expression Signatures of Apoptosis, DNA Damage Response and Unfolded-Protein Response Are Not Affected by GM Feed

**FIGURE 1:**
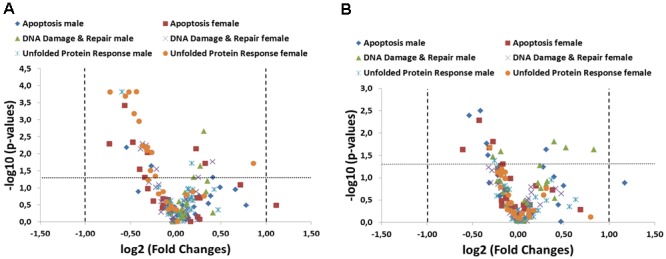
Summary of targeted pathway analysis by RT-qPCR arrays including 79 specific transcripts. A volcano plot analysis of gene expression differences between the 33% GM maize and 33% near-isogenic control maize group is plotted on the *x*-axis (log2 scale), and the statistical significance (*p* < 0.05) is plotted on the *y*-axis (–log10 scale) both in ileum **(A)** and colon **(B)**. The dotted lines indicate fold-changes higher or lower than a twofold up- or down-regulation (values right and left of the vertical lines, respectively) as well as statistical significance (values above the horizontal line).

Gene expression studies addressing the physiologically important pathways of apoptosis, DNA damage and repair and unfolded protein response (UPR) have been introduced in order to evaluate if GM feed has an impact on fundamental signaling pathways associated with cellular stress response and intestinal dysfunction. In the present study, samples were taken from two 90-day studies (the so-called trials A and B; (Zeljenkova et al., 2014)) and 79 marker genes were quantified using qPCR. These marker genes split into 26 genes whose products are involved in apoptosis and UPR pathways and 27 genes whose products are involved in DNA damage and repair (DDR) pathways. Intestinal tissue samples from ileum (**Figure 1A**) as well as colon (**Figure 1B**) of the 33% GMO maize and 33% near-isogenic maize control group of study A were analyzed. We applied the generally accepted threshold of twofold difference for considering qPCR results for differential expression. In both intestinal regions, we observed statistically significant fold changes between the groups. However, those marker genes did not exceed a twofold up- or down-regulation cut-off threshold (**Figures 1A,B**). This applied to 6 genes in ileum of males, 26 genes in female ileum, 11 genes in male colon, and 4 genes in female colon. Individual profiles of marker genes and statistical significances are shown in Supplementary Figures S1, S2 (electronic Supplementary Material). In the case of the apoptosis pathway, the profiles demonstrate that essentially no distinct regulatory pattern was observed. One pro-apoptotic gene (*Fas*) exceeded the cut-off point by a fold change of 2.17 in the female ileal group and a fold change of 2.25 in the male colon group without a statistical significance in both cases. For some marker genes, we observed significant tendencies (less than twofold difference) toward up- or downregulation. However, these genes were well below the twofold cut-off and did not point to co-ordinately differential expression pattern of pro- and anti-apoptotic genes. The analysis of DDR marker genes did not reveal any regulation with respect to the fold change cut-off, while 13 genes showed statistically significant tendencies, which again did not reveal informative patterns of differential expression (Supplementary Figures S1, S2). The UPR pathway analysis revealed no differential expression with respect to the applied fold change cut-off. In female ileum 54% of the studied UPR marker genes showed statistically significant 1.2 – 1.82-fold differences between the study groups, whereby seven of these genes encode heat shock proteins (**Figure 1A** and electronic Supplementary Material 1, Supplementary Figure S1F). However, in male animals as well as in colon tissue no such tendencies were observed (**Figure 1B** and electronic Supplementary Material 1, Supplementary Figures S1E, S2E,F). Taken together, we conclude that only minor tendencies, which do not reflect distinct perturbations of the studied pathways, were observed.

### Comprehensive Analysis of the Transcriptional Response in Rat Intestine to GM Feed

To assess if feeding GM-maize affects global gene expression in the rat small intestine, we compared RNAseq based expression profiles of rat ileum from both study groups receiving 33% GM or near-isogenic control maize. In total, we detected 34.276 (study A) and 25.570 (study B) transcripts by the RNAseq profiling. In study A, we identified 102 transcripts to be at least twofold up-or down regulated in GM maize-fed male animals, while in female intestinal tissues, 63 transcripts showed altered concentrations comparing both study groups (Log2 fold-change > 1 or < -1, *q*-value < 0.05, false discovery rate [FDR] < 5%; see electronic Supplementary Material 1, Supplementary Figures S3A,C). In study B, we observed a differential expression of 30 and 18 transcripts in males and females, respectively (Log2 fold-change > 1 or < -1, *q*-value < 0.05, false discovery rate [FDR] < 5%; see electronic Supplementary Material 1, Supplementary Figures S3B,C). In a comparative analysis, we identified 18 commonly differentially expressed transcripts in males after matching both studies A and B, while 12 transcripts overlapped in datasets of differentially expressed genes comparing females of both studies A and B (electronic Supplementary Material 1, Supplementary Figure S3C).

### Global Transcriptional Profiles Point to Intestinal Circadian Rhythm

**FIGURE 2:**
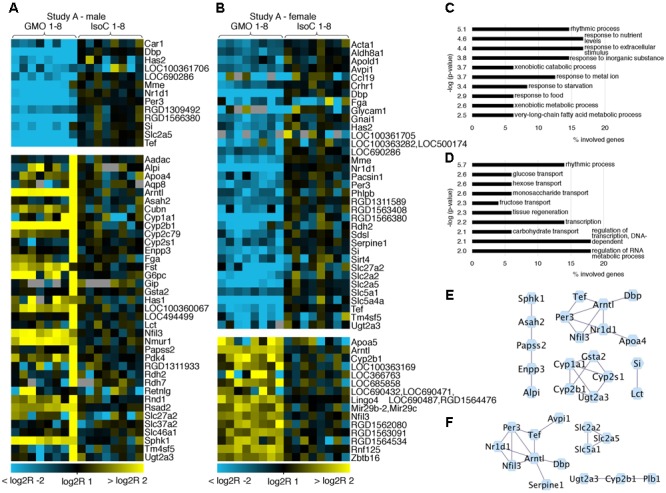
Heatmap of differentially expressed genes and gene ontology (GO) analysis. Heatmaps showing the top 50 differentially expressed genes with more than twofold up- or down-regulation (*q* < 0.05) in male **(A)** and female **(B)** animals of study A. GO analysis of the differentially expressed genes (log2R > 1 or < –1) indicates pathways in which regulated genes show a highly significant enrichment in males **(C)** or females **(D)**. Networks of protein-protein interactions of males **(E)** or females **(F)** using STRING demonstrate associations in sets of differentially expressed genes.

**FIGURE 3:**
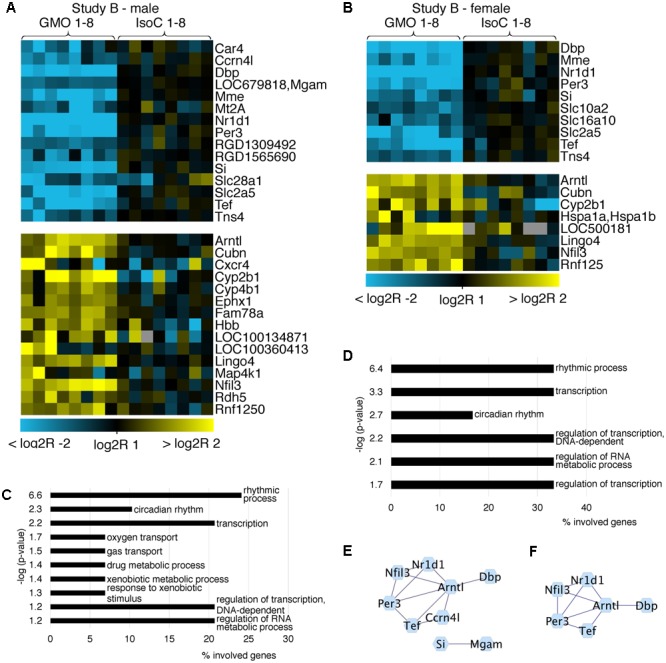
Heatmap of differentially expressed genes and GO analysis. Heatmaps showing all differentially expressed genes with more than a twofold up- or down-regulation (*q* < 0.05) in male **(A)** and female **(B)** animals of study B. GO analysis **(C,D)** of the differentially expressed genes (log2R > 1 or < –1) indicates pathways in which regulated genes show a highly significant enrichment in males **(C)** or females **(D)**. Networks of protein-protein interactions of males **(E)** or females **(F)** showing associations in sets of differentially expressed genes.

**FIGURE 4:**
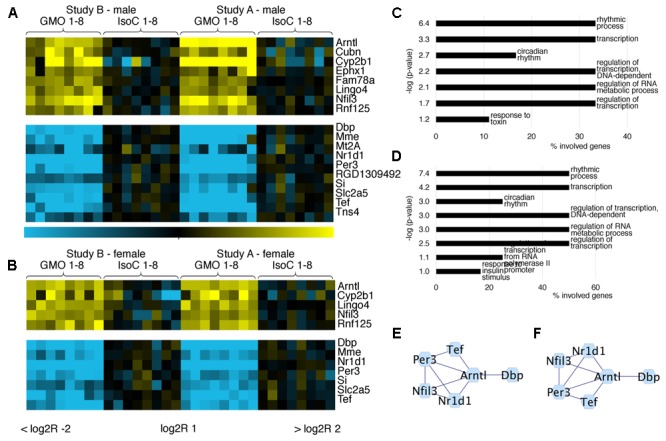
Comparative analysis of commonly differentially expressed genes in both feeding trials. Heatmaps showing the top differentially expressed genes with more than twofold up/down-regulation (*q* < 0.05) in ileum of male **(A)** and female **(B)** rats fed near-isogenic or GM maize. GO analysis of differentially expressed genes (log2R > 1 or < –1) in males **(C)** or females **(D)** indicates pathways with significantly regulated genes. Networks of protein-protein interactions in males **(E)** or females **(F)** showing associations in sets of differentially expressed genes.

In both studies A and B 0.3–0.8% of all transcripts detected showed significant differences between the GMO and control groups (Supplementary Figures S3A,B). To determine its biological meaning, we performed an enrichment analysis of GO annotated biological processes using DAVID (Huang da et al., 2009) and applied a network analysis using STRING (Franceschini et al., 2013) and Cytoscape (Shannon et al., 2003). In study A, it was apparent, that the list of genes with the highest fold changes included various members of the circadian clock pathway (**Figures 2A,B**). For example, in males of study A, all transcripts being more than sixfold down regulated in the GMO group (*Per3, Nr1d1, Tef*, and *Dbp*) are connected with the pathway of circadian rhythms, while genes showing the highest up-regulation point to the relevance of metabolic processes as revealed by GO-analysis. For both male and female animals of study A, the top 50 transcripts with a more than twofold difference between the study groups are shown in **Figures 2A,B**. Throughout both animal trials and both sexes, the top ten biological processes associated with differentially expressed genes (Log2 fold-change > 1 or < -1, *q*-value ≤ 0.05) pointed to circadian rhythms, and in all cases, the highest significance in the enrichment of genes was observed for “rhythmic process” (**Figures 3, 4C,D**). Consequently, further biological processes included metabolic processes and nutrient transport, which are well known to be strongly affected by circadian rhythms (Green et al., 2008; Hussain and Pan, 2009; Hussain, 2014). In line with the potential functional interactivity between transcripts that showed differential expression between study groups, we performed a network analysis, which revealed connected elements that are known to interact (based on Text mining, Experiments and Databases). The interaction network nodes with most direct interactions always pointed to the circadian clock pathway, as shown in **Figure 2** for study A males (e) and females (f), as well as study B males and females (**Figures 3E,F**). In **Figure 4**, the commonly differentially expressed transcripts comparing studies A and B are shown, thereby underlining the consistency of observed perturbations in circadian pathways in both animal trials.

### Differentially Expressed Genes Are Related to Circadian Regulation

**FIGURE 5:**
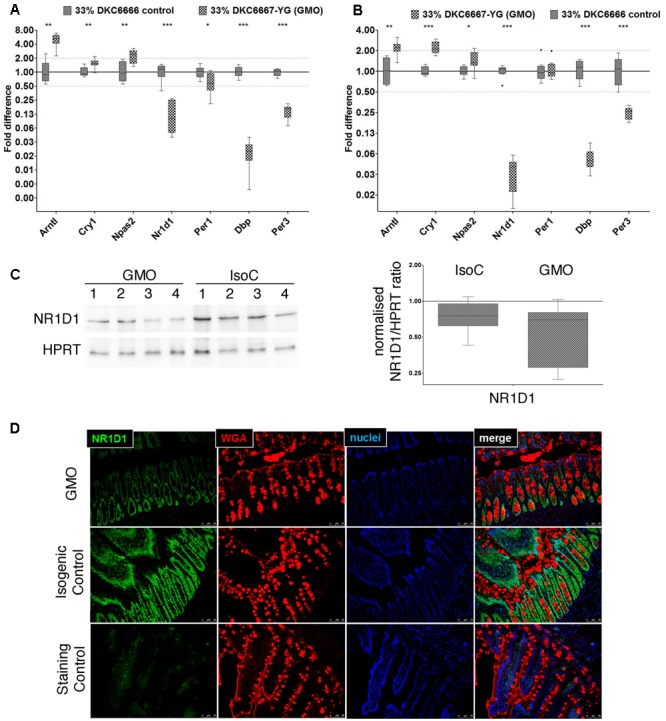
Differential expression of selected core circadian clock genes and NR1D1 protein in ileum. RT-qPCR analysis of circadian clock genes in male **(A)** and female **(B)** rats from study A. **(C)** Representative Western Blot images of NR1D1 in female ileum (left), and densitometric analysis of three replicates of Western blot analyses and of eight biological replicates from each study group (right). When normalized for HPRT protein expression, there was no significant difference between the study groups **(D)** Immunostaining of NR1D1 protein (green) in small intestinal paraffin embedded tissue sections demonstrates nuclear localization in intestinal epithelial cells (representative image). Wheat germ agglutinin (WGA) immunostaining (red) was used for outlining cell borders and mucus; the blue color indicates staining of nuclei by 4’,6-diamidino-2-phenylindole (DAPI). ^∗^*P* < 0.05; ^∗∗^*P* < 0.01; ^∗∗∗^*P* < 0.001; Mann–Whitney *U*-test.

In order to confirm our hypothesis that differences in gene expression between study groups in both studies A and B are related to the time of sampling, we performed RT-qPCR analysis of seven genes centrally involved in circadian rhythms. First, we technically compared fold differences observed using RNAseq and RT-qPCR analyses and observed a good correlation of differential expression values (Spearman’s rank correlation coefficient: 0.94), while the RT-qPCR approach tended to generate higher total fold differences (electronic Supplementary Material 1, Supplementary Figure S4). Using the RT-qPCR approach, we observed strong up regulation of the circadian clock gene *Bmal1* (*Arntl*) as well as a strong down regulation of *Dbp, Nr1d1* (*rev-Erbα*), and *Per3* in male and female ileal tissues and in both studies (**Figures 5A,B**). Likewise, we detected similar effects in the male and female colonic tissues (electronic Supplementary Material 1, Supplementary Figure S5). Here, the mRNAs of *bmal, dbp, nr1d1*, and *per3* showed the same regulation as in the ileum, and cry1 was in addition significantly upregulated. Nr1d1 exists in two slightly different isoforms: *rev-Erbα1* and *rev-Erbα2* (Rambaud et al., 2009). A detailed analysis of these two isoforms in the female ileum via RT-qPCR revealed a comparable circadian regulation. However, we observed a notable difference in the expression of the isoforms. *Rev-Erbα1* showed a 40–60-fold higher expression compared to *rev-Erbα2* (data not shown). Quantification of Nr1d1 protein in female ileal samples resulted in a statistically non-significant 21% down-regulation between the GMO and the isogenic control group (*p* = 0.117) (**Figure 5C**). Furthermore, *Nr1d1* expression was localized in the nuclei of small intestinal epithelial cells as assessed by immunostaining of paraffin sections of small intestine tissue samples (**Figure 5D**). Accordingly, a tendency toward a lower protein expression was observed in the GMO groups.

### Genes Associated with Colorectal Cancer Do Not Show Altered Expression Levels between the Study Groups

We compared our data sets with a gene set from TissGDB, which is tissue-specific gene annotation database in cancer^2^ (Kim et al., 2017). The RNAseq based expression profiles from both studies A and B included 65 of the 80 reference genes for colorectal cancer. In both male as well as in female animals, there was a near-complete absence of significantly altered gene expression levels in the GMO-group compared to controls (Log2 fold-change > 1 or < -1, *q*-value < 0.05, false discovery rate [FDR] < 5%; see electronic Supplementary Material 1, Supplementary Figure S6). However, there was an exception in male rats in study A. Here, we observed a significantly 2.8- and 4.3-fold higher expression of Cyp2s1 and Agp8 in the GMO-group compared to controls, respectively.

## Discussion

The UPR pathway reflects cellular stress related to the endoplasmic reticulum (ER) and is affected in many diseases (Zhang and Kaufman, 2008; Adolph et al., 2013). Apoptosis is essential for the maintenance of normal gut epithelial function, and deregulation of apoptosis occurs in a number of pathological conditions in the gastrointestinal tract (Ramachandran et al., 2000). DNA damage response signaling is another fundamental pathway affecting human and animal diseases, such as intestinal cancer (Jackson and Bartek, 2009). When investigating rat ileal and colon tissues after a 90 days GM-maize feeding study, the targeted RT-qPCR approach demonstrated some marker genes of the apoptosis, UPR and DNA-damage and repair pathways reaching either a statistical significance or exceeding the twofold change threshold. Strikingly, no measured individual mRNA fulfilled requirements for both statistical significance and fold-change cut-off that would point toward a biological relevance. The Cell Surface Death Receptor (Fas) was the only gene, which exceeded the twofold qPCR threshold. However, the inter-individual variation for this factor in the tissue samples led to a high *p*-value and consequently to no statistical significance between the control and the GM-maize fed groups. The highest accumulation of statistical significant genes (13 of 27 genes) appeared in the UPR pathways of the female ileum. However, there was no analog observation in the analyzed male ileal and colonic as well as in the female colonic tissue. In summary, there is no verifiable and reproducible effect on ileal and colonic tissue in both genders when comparing the feeding groups.

Analysis of the RNA-seq data revealed that most of the transcriptome remained unchanged. However, we detected changes that are presumably linked to the experimental design, such as the time of sampling and accordingly the fasting period. Differentially expressed genes reflected distinct pathways of circadian rhythms, which in turn drive the rhythmic expression of a wide variety of genes that orchestrate metabolism and the immune response.

The comparison of the RNAseq based data with a gene set for colorectal cancer (Kim et al., 2017) did not point to cancer-specific expression pattern. The only genes showing higher expression levels in GMO-treated males of study A belonged to the group of aquaporins (Agp8) and to the cytochrome P450 gene family (Cyp2s1). It is long established that the expression of cytochrome P450 enzymes is under circadian control (Froy, 2009). Also, a number of aquaporins seem regulated by molecular clocks. For example, in mouse salivary glands as well as in mouse dorsal skin, circadian oscillation of aquaporin was observed (Matsunaga et al., 2014; Uchida et al., 2017).

The intestine is known to be an important organ for circadian rhythms of gene expression and key factors of the mammalian circadian clock have previously been deciphered. Arntl and Npas2 (an ortholog of Clock) form a DNA-binding heterodimer. The binding of Arntl:Npas2 leads to the expression of Dbp and subsequently of that of Cry1/2, and Per1/2/3. The heterodimer formed by Per and Cry proteins represses in a negative feedback loop the expression of Arntl and Npas2. The second negative feedback loop is the induced expression of Nr1d1 by Arntl:Npas2 while Nr1d1 represses again its own inducers. The peripheral circadian rhythm in the intestine is mediated by an endocrine signaling controlled by the central clock on the one hand and by the organism’s food intake on the other hand (Stokkan et al., 2001).

We chose Nr1d1 as a representative factor for the circadian regulation in the ileum and analyzed gene expression and protein abundance in detail. Consistent with our NGS data, the RT-qPCR array as well as the specific NR1D1-isoform RT-qPCR analysis pointed to a strong differential expression between the two analyzed groups. Only the quantification of the protein showed a slight difference between the groups. This may be explained by a potential discrepancy between pure mRNA expression data and protein translation as well as post-translational modifications and protein degradation. It has previously been described that alternative promoter usage may lead to the isoform rev-erbα1 bearing a PEST sequence in the 5′-region of the mRNA while rev-erbα2 exhibits this feature (Triqueneaux 2004). The consequences are two proteins, one of them exposed to degradation processes via phosphorylation and the other one without the phosphorylation site with a higher stability. The turnover of mRNAs and the translation, modification and also degradation of proteins are not necessarily linear. Given the unequally stable rev-erbα isoforms it is most likely that the amount of mRNA does not reflect the protein concentration of the corresponding protein in the cell. The rather low downregulation of the NR1D1 protein in the ileum compared to the RT-qPCR data could therefore be due to the post-transcriptional modifications.

Bioinformatics analysis indicated that differentially expressed transcripts are under substantial circadian control in the feeding trials. In randomized as well as block experimental designs this is important, since these transcripts may substantially vary, depending on different daytimes, and will produce variation in the treatment groups. A documentation of sampling times is strongly advised in order to assign potential circadian regulation to observed effects in selected tissues.

## Conclusion

Comprehensive transcriptional profiles of rat intestinal tissue in the frame of two subchronic (90-day) GM-maize feeding trials (Zeljenkova et al., 2014) were analyzed, which facilitated a holistic view of potentially affected intestinal signaling pathways. By bioinformatics analysis of differentially expressed genes, significant differences between the study groups could clearly be attributed experimental design issues, especially to the time of sampling. This underlines the potential of RNAseq approaches to identify biological differences between study groups by allowing complex mechanistic insights into intestinal signaling. In addition, an interpretation of the obtained results enabled hypothesis generation (e.g., the effect of the sampling time on circadian clock genes). The observed absence of perturbation of pathways that could point to intestinal toxicity may therefore increase confidence in the study results revealed by common OECD endpoints. However, a thorough validation of the predictive potential of transcriptomic studies to evaluate intestinal toxicity requires adequate positive controls of known and quantifiable toxicity *in vivo*. Subchronic feeding trials with whole GM maize lack targeted hypotheses, and adequate positive controls – for unforeseen effects – are not available, as most of the frequently published feeding studies never showed deleterious effects when feeding commercialized GM material to animals (for reviews see (Snell et al., 2012; Van Eenennaam and Young, 2014).

## Author Contributions

JS, MB, NB, and DZ have carried out all animal studies. JS, MB, and NB have carried out the expression studies. AK, CB, and AF have performed the NGS and bioinformatic analysis. RE and PS have planned and supervised the study. JS and RE have written the manuscript.

## Conflict of Interest Statement

The authors declare that the research was conducted in the absence of any commercial or financial relationships that could be construed as a potential conflict of interest.

## Footnotes

1 http://NCBI.nlm.nih.gov/geo/

2 http://zhaobioinfo.org/TissGDB
