# Supplementary material for: Transcriptomic Analysis of Intestinal Tissues from Two 90-Day Feeding Studies in Rats Using Genetically Modified MON810 Maize Varieties
Source: Front Genet. 2017 Dec 19;8:222. doi: 10.3389/fgene.2017.00222 (PMC5742243; doi:10.3389/fgene.2017.00222)
Supplement: Supplementary file 1 [file Table_1.PDF]

**Transcriptomic analysis of intestinal tissues from two 90-day feeding studies in rats using genetically modified MON810 maize varieties**

Jutta Sharbati, Marc Bohmer, Nils Bohmer, Andreas Keller, Christina Backes, Markus Schilhabel, Dagmar Zeljenková, Ralf Einspanier

Supplementary Table 1: Oligonucleotides used in this study

| Primerpair Nr. | Gene name | Accession number | Oligo name | Sequence 5'-3' | Product size bp | T an opt | Comments | PCR Efficiency | r² of calibration curve | Linear dynamic range |
|---|---|---|---|---|---|---|---|---|---|---|
| 1 | Abl1 | NM_001100850 | rn_abl1_fw | ATC TCG CTG CGG TAT GAA GG | 194 | 60 | intron seperated | 95.73 | 0.999 | 7 logs |
| | | | rn_abl1_rev | CCG TAG ATG GTG GGC TTG TT | | | exon3-exon4 | | | |
| 2 | Actb | NM_031144 | rn_actb_fw | CGC GAG TAC AAC CTT CTT GC | 211 | 60 | intron seperated | 93.24 | 0.998 | 8 logs |
| | | | rn_actb_rev | CCT TCT GAC CCA TAC CCA CC | | | exon1-exon3 | | | |
| 3 | Akt1 | NM_033230 | rn_akt1_fw | TCA AGA TGA CAG CAT GGA GTG TG | 216 | 60 | intron seperated | 95.99 | 0.998 | 8 logs |
| | | | rn_akt1_rev | AAT TCC GAG GCT GGC GAG G | | | exon12-exon13 | | | |
| 4 | Apex1 | NM_024148 | rn_apex1_fw | CTC GTT GGG AGG CAG CGT AG | 153 | 60 | intron seperated | 98.86 | 0.999 | 7 logs |
| | | | rn_apex1_rev | CTG CCC CCT TAC TCT TCT TGG | | | exon1-exon3 | | | |
| 5 | Artnl | NM_024362.2 | rn_arntl_fw | AAT GAC TGT CTA GGT GGA GGA TT | 173 | 60 | intron seperated | 83.84 | 0.997 | 7 logs |
| | | | rn_arntl_rev | CAC CCT CTG GGC CCA AAT TC | | | exon3-exon5 | | | |
| 6 | Atf4 | NM_024403 | rn_atf4_fw | GAA GCC TGA CTC TGC TGC TT | 196 | 60 | | 103.94 | 0.999 | 8 logs |
| | | | rn_atf4_rev | TTT GGG TCG AGA ACC ACG AG | | | exon3 | | | |
| 7 | Atf6 | NM_001107196 | rn_atf6_fw | TGA ACT TCG AGG CTG GGT TC | 208 | 60 | intron seperated | 97.35 | 0.999 | 8 logs |
| | | | rn_atf6_rev | ACT TCC AGG CGA AGC GTA AT | | | exon1-exon3 | | | |
| 8 | B2m | NM_012512 | rn_b2m_fw | AGG TGA AGA GGC CTA GCT GA | 219 | 60 | | 95.09 | 0.999 | 8 logs |
| | | | rn_b2m_rev | GGG TGA TGA AAA CCG CAC AC | | | exon4 | | | |
| 9 | Bad | NM_022698 | rn_bad_fw | GCA GCC CAG AGT ATG TTC CA | 213 | 60 | intron seperated | 98.06 | 0.996 | 7 logs |
| | | | rn_bad_rev | ACT CCG GGT CTC CAT AGT CC | | | exon1-exon3 | | | |
| 10 | Bcl2 | NM_016993 | rn_bcl2_fw | CTG GTG GAC AAC ATC GCT CT | 206 | 60 | intron seperated | 96.26 | 0.998 | 7 logs |
| | | | rn_bcl2_rev | CCC AGG TAT GCA CCC AGA GT | | | exon1-exon2 | | | |
| 11 | Bcl2l1 | NM_031535 | rn_bcl2l1_fw | CAG GAC TGA AGC CCC AGA AG | 196 | 60 | | 101.75 | 0.996 | 8 logs |
| | | | rn_bcl2l1_rev | TTC AAA CTC ATC GCC AGC CT | | | exon2 | | | |
| 12 | Bid | NM_022684 | rn_bid_fw | TAG AGC TCG GGT CCA AGT GT | 182 | 60 | intron seperated | 90.00 | 0.998 | 7 logs |
| | | | rn_bid_rev | TTC GGA GAA AGC CGA ACA CC | | | exon1-exon3 | | | |
| 13 | Birc2 | NM_021752 | rn_birc2_fw | TTT GCC TGT GGT GGG AAA CT | 180 | 60 | | 96.71 | 0.998 | 8 logs |
| | | | rn_birc2_rev | TGG CCA GTA CAG AAA CGT CC | | | exon1 | | | |
| 14 | Birc3 | NM_023987 | rn_birc3_fw | ATG GAC AAG GAC AGT GAG CG | 218 | 60 | intron seperated | 94.43 | 0.997 | 7 logs |
| | | | rn_birc3_rev | TTC ATC AGC TTG GCC AGG AA | | | exon2-exon3 | | | |
| 15 | Birc5 | NM_022274 | rn_birc5_fw | GAG CAT AGG AAG CAC TCC CC | 214 | 60 | intron seperated | 97.01 | 0.997 | 7 logs |
| | | | rn_birc5_rev | CAG CAA AGG CTC AGC GTA AG | | | exon3-exon4 | | | |
| 16 | Brca2 | NM_031542 | rn_brca2_fw | GTG AAA AAT GCA GGT TTA ATA ACC A | 220 | 60 | intron seperated | 92.84 | 0.997 | 7 logs |
| | | | rn_brca2_rev | TGC TTC TTT TGA TTG AAG AAT CTG G | | | exon10-exon11 | | | |
| 17 | Casp12 | NM_130422 | rn_casp12_fw | TCG GAG AAG GAG CGA GCT TA | 207 | 60 | intron seperated | 100.04 | 0.99 | 8 logs |
| | | | rn_casp12_rev | CTT TTC CCC TGG ATT CTG ATG C | | | exon2-exon4 | | | |
| 18 | Casp3 | NM_012922 | rn_casp3_fw | GGA GCT TGG AAC GCG AAG A | 220 | 60 | intron seperated | 106.81 | 0.995 | 8 logs |
| | | | rn_casp3_rev | GCG AGC TGA CAT TCC AGT GC | | | exon1-exon4 | | | |
| 19 | Casp6 | NM_031775 | rn_casp6_fw | CTC GTG GGC AGC CTG AAA AG | 215 | 60 | intron seperated | 100.48 | 0.996 | 8 logs |
| | | | rn_casp6_rev | TTC CCT ACT TCT GTA GAA GCC AT | | | exon1-exon4 | | | |
| 20 | Casp7 | NM_022260 | rn_casp7_fw | CAC CAT GGG GCT GAA GAG TT | 180 | 60 | | 101.53 | 0.998 | 8 logs |
| | | | rn_casp7_rev | GGG AGG TCT GGG GGT ATC TT | | | exon6 | | | |
| 21 | Casp8 | NM_022277 | rn_casp8_fw | GTA AAC TTT GGC GGA CTG GC | 214 | 60 | intron seperated | 102.24 | 0.997 | 7 logs |
| | | | rn_casp8_rev | TTC TTC GTT GCC CAG ACG TT | | | exon1-exon2 | | | |
| 22 | Casp9 | NM_031632 | rn_casp9_fw | CTG TCC CGT GAA GCA AGG AT | 220 | 60 | | 97.56 | 0.998 | 8 logs |
| | | | rn_casp9_rev | TGG TAC ATC GGC AGA GAA GC | | | exon9 | | | |
| 23 | Cry1 | NM_198750.2 | rn_cry1_fw | ATG CTC CTG GAG AGA ATG TCC | 240 | | intron seperated | 85.74 | 0.999 | 7 logs |
| | | | rn_cry1_rev | CCT CCC GCA TGC TTT CGT ATC | | | exon10-exon13 | | | |
| 24 | Cycs | NM_012839 | rn_cycs_fw | TTT GTT GGA CAG CCC CGA TT | 180 | 60 | | 103.27 | 0.997 | 8 logs |
| | | | rn_cycs_rev | ATA GGT TTG AGG CGA CAC CC | | | exon3 | | | |
| 25 | Dbp | NM_012543.3 | rn_dbp_fw | TTT CGG GGA CGT GGA ATA CG | 249 | 60 | intron seperated | 78.44 | 0.999 | 7 logs |
| | | | rn_dbp_rev | GGA CTG GGT GTG TCC CTA GA | | | exon2-exon3 | | | |
| 26 | Dclre1a | NM_001106201 | rn_dclre1a_fw | AGG TTC AAA GGT GGG CAT GT | 207 | 60 | intron seperated | 95.74 | 0.999 | 8 logs |
| | | | rn_dclre1a_rev | CCT GTA GGT CGG AAA GCC AA | | | exon7-exon8 | | | |
| 27 | Ddb1 | NM_171995 | rn_ddb1_fw | TCT ACC AGG ATC CTC AGG GG | 215 | 60 | intron seperated | 95.52 | 0.997 | 7 logs |
| | | | rn_ddb1_rev | GAT AGG AGG AGC GAT TGC CA | | | exon4-exon6 | | | |

| | | | | | | | | | | |
|---|---|---|---|---|---|---|---|---|---|---|
| 28 | Ddb2 | NM_001271346 | rn_ddb2_fw<br>rn_ddb2_rev | GCT CCC AAG AAA TGC CCA GA<br>GCA GCA CGA CTC ACA TTT CC | 153 | 60 | intron seperated<br>exon1-exon2 | 98.10 | 0.999 | 7 logs |
| 29 | Dffa | NM_053679 | rn_dffa_fw<br>rn_dffa_rev | AGG GCA GCA TCT TGT CCA AC<br>GTT CCT AGT TCT TGC CCA CCT | 198 | 60 | intron seperated<br>exon4-exon5 | 105.20 | 0.999 | 8 logs |
| 30 | Dffb | NM_053362 | rn_dffb_fw<br>rn_dffb_rev | TAC CCC AGA AGG CTG GTT CT<br>CTC CCG TCC TGG ATT GCT TC | 186 | 60 | intron seperated<br>exon5-exon7 | 93.61 | 0.999 | 8 logs |
| 31 | Dnajb9 | NM_012699 | rn_dnajb9_fw<br>rn_dnajb9_rev | AAC AGG ACG AAG GTT GCT CG<br>AAC TGA CTG TGG AGT TGC CA | 168 | 60 | intron seperated<br>exon1-exon2 | 100.68 | 0.999 | 8 logs |
| 32 | Dnajc10 | NM_001106486 | rn_dnajc10_fw<br>rn_dnajc10_rev | GCA GTG AAG TAC AAC GGG GA<br>CTA AGC CGG AGT CGT GTC TG | 203 | 60 | intron seperated<br>exon7-exon9 | 113.16 | 0.999 | 8 logs |
| 33 | Dnajc3 | NM_022232 | rn_dnajc3_fw<br>rn_dnajc3_rev | AGA GAA AGC CCA GCG GTT AC<br>TGG GTC GGA GAG GAC TTC TT | 217 | 60 | intron seperated<br>exon10-exon11 | 110.60 | 0.999 | 8 logs |
| 34 | Edem1 | XM_238366 | rn_edem1_fw<br>rn_edem1_rev | ACA GGG ATC CCC TAT CCT CG<br>CGA AGG TTC CAG AGG GCT TT | 182 | 60 | intron seperated<br>exon4-exon6 | 95.04 | 0.999 | 8 logs |
| 35 | Edem2 | NM_001004230 | rn_edem2_fw<br>rn_edem2_rev | TCG AGA GCG CAA TGT ACC TC<br>ATT TCA CAG TCT CGG CCA GG | 186 | 60 | intron seperated<br>exon10-exon11 | 106.25 | 0.999 | 8 logs |
| 36 | Eif2a | NM_001109339 | rn_eif2a_fw<br>rn_eif2a_rev | ACA AGA CAG GCG CTT CCT AC<br>CGA GGA CCA GTC CCA AAG TC | 220 | 60 | intron seperated<br>exon9-exon10 | 108.25 | 0.998 | 8 logs |
| 37 | Ero1l | NM_138528 | rn_ero1l_fw<br>rn_ero1l_rev | AGC GGT GTT TCT GTC AGG TT<br>GAA TGG CAG GGT TTG ACA GC | 220 | 60 | intron seperated<br>exon1-exon3 | 103.68 | 0.999 | 8 logs |
| 38 | Exo1 | NM_001107198 | rn_exo1_fw<br>rn_exo1_rev | AGC CTT GCG TGT TGA AGA GA<br>TTG TGA GCA GTC CCA CCA TC | 169 | 60 | intron seperated<br>exon10-exon11 | 88.18 | 0.999 | 8 logs |
| 39 | Fadd | NM_152937 | rn_fadd_fw<br>rn_fadd_rev | GCC TGA GTG ATC GGG TAA GG<br>CAC AGT TGA ATC CCT CAG CG | 200 | 60 | exon2 | 103.32 | 0.997 | 7 logs |
| 40 | Fas | NM_139194 | rn_fas_fw<br>rn_fas_rev | ATG GTC TGG GAG GAC CAT GA<br>TGG CAA AAA GAA CAC GCC AG | 186 | 60 | exon9 | 108.06 | 0.998 | 8 logs |
| 41 | Faslg | NM_012908 | rn_faslg_fw<br>rn_faslg_rev | GCA TCA TGA GCC AGA TGG GA<br>AAA AGA CGG CCT CCT GTG AG | 199 | 60 | exon4 | 102.00 | 0.999 | 8 logs |
| 42 | Fen1 | NM_053430 | rn_fen1_fw<br>rn_fen1_rev | CGA ACA TCT CTC TTC GCT GGT<br>ATC AAT GGC CAC TTT GCG AC | 151 | 60 | intron seperated<br>exon1-exon2 | 93.89 | 0.999 | 8 logs |
| 43 | Hprt1 | NM_012583 | rn_hprt1_fw<br>rn_hprt1_rev | GCC TAA AAG ACA GCG GCA AG<br>GGC TGC CTA CAG GCT CAT AG | 198 | 60 | exon9 | 97.76 | 0.999 | 8 logs |
| 44 | Hsp90b1 | NM_001012197 | rn_hsp90b1_fw<br>rn_hsp90b1_rev | TAA GCT CTA TGT GCG CCG AG<br>CCA GAG TTT TGC GGA CAA GC | 182 | 60 | intron seperated<br>exon10-exon12 | 103.14 | 0.995 | 8 logs |
| 45 | Hspa2 | NM_021863 | rn_hspa2_fw<br>rn_hspa2_rev | TCC TAA CGT TGC TTT GCC TG<br>TCA TGG CCA CTT GGT TCT TG | 220 | 60 | | 102.72 | 0.997 | 6 logs |
| 46 | HSPA4 | NM_153629 | rn_hspa4_fw<br>rn_hspa4_rev | AGT GCG AAT GCT TCA GAC CT<br>CGT GTG GCT CCA CCA ACT AT | 206 | 60 | intron seperated<br>exon7-exon9 | 110.59 | 0.999 | 8 logs |
| 47 | Hspa4l | NM_001106428 | rn_hspa4l_fw<br>rn_hspa4l_rev | CAC GCC GGC CTG TAT ATC TT<br>GGG AAG CCT GAT TCT CTC CG | 163 | 60 | intron seperated<br>exon3-exon4 | 106.25 | 0.999 | 8 logs |
| 48 | Hspa5 | NM_013083 | rn_hspa5_fw<br>rn_hspa5_rev | CTA CGA AGG TGA ACG ACC CC<br>ATT TCT TCA GGG GTC AGG CG | 219 | 60 | exon9 | 99.76 | 0.997 | 8 logs |
| 49 | Hspb9 | NM_001108835 | rn_hspb9_fw<br>rn_hspb9_rev | GTA GCT TCC CGA TGT CCC AG<br>TTC TGG CCA TCT ATC CGC AC | 188 | 60 | | 103.80 | 0.997 | 7 logs |
| 50 | Hsph1 | NM_001011901 | rn_hsph1_fw<br>rn_hsph1_rev | ACG TCA GTA CCA CGC TCA AC<br>TTC CGA CTG AAC ACC TCG TG | 187 | 60 | intron seperated | 101.65 | 0.998 | 8 logs |
| 51 | Ldha | NM_017025 | rn_ldha_fw<br>rn_ldha_rev | TGC ACT AAG CGG TCC CAA AA<br>GCA AGC TCA TCA GCC AAG TC | 181 | 60 | intron seperated<br>exon1-exon3 | 94.32 | 0.994 | 8 logs |
| 52 | Lig1 | NM_001024268 | rn_lig1_fw<br>rn_lig1_rev | TTT CGA GTT TGC GTC TCC GA<br>GGA GGG GGT TCC TTC TCT CT | 219 | 60 | intron seperated<br>exon1-exon3 | 98.67 | 0.998 | 8 logs |
| 53 | Lig3 | NM_001012011 | rn_lig3_fw<br>rn_lig3_rev | AGC GGG TCC TTC ACA ATG AG<br>TGC GGC TGA AGT AGC TGA AA | 231 | 60 | intron seperated<br>exon8-exon9 | 89.48 | 0.997 | 7 logs |
| 54 | Lig4 | NM_001106095 | rn_lig4_fw<br>rn_lig4_rev | CGG CTG GAC TGG GAT TTA CA<br>TGC ACG GTC TTT ACC TTT CTG T | 158 | 60 | intron seperated<br>exon1-exon2 | 96.35 | 0.998 | 8 logs |
| 55 | Mapk1 | NM_053842.1 | rn_mapk1_fw<br>rn_mapk1_rev | TGC TCG ATT CCA GCC AGG AT<br>GCC ACT ACG ACC AGA CTG C | 180 | 60 | intron seperated<br>exon8-exon9 | 110.88 | 0.999 | 8 logs |
| 56 | Mlh1 | NM_031053 | rn_mlh1_fw<br>rn_mlh1_rev | CCA TGT GGC CCA TGT CAC TA<br>TCC CGT ACT CTT CAC TGG GAT | 200 | 60 | intron seperated<br>exon5-exon6 | 90.49 | 0.997 | 8 logs |
| 57 | Mlh3 | NM_001108043 | rn_mlh3_fw | TGG AGG TGG TTA GTC CGT GA | 155 | 60 | intron seperated | 95.53 | 0.999 | 8 logs |

| | | | | | | | | | | |
|---|---|---|---|---|---|---|---|---|---|---|
| | | | rn_mlh3_rev | AAA CCA GAA CGC AAC TTG GC | | | exon1-exon2 | | | |
| 58 | Msh2 | NM_031058 | rn_msh2_fw<br>rn_msh2_rev | CCG CTT TGG CCA GTC AGA TA<br>TTC TGC TTG GCG CAC TCT AT | 178 | 60 | intron seperated<br>exon14-exon15 | 91.51 | 0.999 | 8 logs |
| 59 | Msh3 | NM_001191957 | rn_msh3_fw<br>rn_msh3_rev | TGG GGA ATT ACC ACA TGG GC<br>TGC TAG CTT CGC CAC ATT CA | 155 | 60 | intron seperated<br>exon22-exon23 | 87.98 | 0.999 | 8 logs |
| 60 | Nfkb1 | NM_001276711 | rn_nfkb1_fw<br>rn_nfkb1_rev | TTC AAC ATG GCA GAC GAC GA<br>GCA CAC GTA GCG GAA TCG AA | 183 | 60 | intron seperated<br>exon3-exon6 | 104.62 | 0.997 | 7 logs |
| 61 | Npas2 | NM_001108214.2 | rn_npas2_fw<br>rn_npas2_rev | GCA CTT GAT GCA GTT TGG CA<br>CAT GGC TTC TGT GGG TGG AT | 220 | 60 | intron seperated | 88.41 | 0.997 | 8 logs |
| 62 | Nr1d1<br>(isoform1) | NM_001113422.1 | rn_nr1d1α_fw<br>rn_nr1d1α_rev | TGA CGA CCC TAG ACT CCA ACA<br>GTG AGG GAG CCA GTA GGT GA | 181 | 60 | intron seperated | 95.48 | 0.999 | 8 logs |
| 63 | Nr1d1<br>(both isoforms) | NM_145775.2 | rn_nr1d1_iso_fw<br>rn_nr1d1_iso_rev | TCA CGG CAG TGG TAC TTG TC<br>CAG CTT GAG CAG CAG TTT GG | 158 | 60 | | 97.28 | 0.998 | 9 logs |
| 64 | Nr1d1<br>(isoform2) | NM_145775.2 | rn_nr1d1β_fw<br>rn_nr1d1β_rev | TGT TTC TCC CTC TCA CCT CCT<br>GAT CCG CTG GAG CCA ATG TA | 202 | 60 | | 97.62 | 0.999 | 9 logs |
| 65 | Nthl1 | NM_001105728 | rn_nthl1_fw<br>rn_nthl1_rev | TTG CTG GGC AGA CTC ATC TAC<br>GTC CAC TTC AGT CTG TTG GC | 245 | 60 | intron seperated<br>exon3-exon5 | 93.06 | 0.999 | 8 logs |
| 66 | Os9 | NM_001007265 | rn_os9_fw<br>rn_os9_rev | TGG GAC GAT GAA ACA GCC AA<br>CCT CGA TGT CCG AAT GCT CA | 219 | 60 | intron seperated<br>exon4-exon6 | 109.73 | 0.999 | 8 logs |
| 67 | Parp1 | NM_013063 | rn_parp1_fw<br>rn_parp1_rev | GGA CAA CCT CCT GGA CAT CG<br>TCA TAG GCA TTG TGC GTG GT | 195 | 60 | intron seperated<br>exon17-exon18 | 85.79 | 0.999 | 8 logs |
| 68 | Parp2 | NM_001106030 | rn_parp2_fw<br>rn_parp2_rev | GAG ATC TGG CTG TGG AAG ACG A<br>CCT TTT GTC CTG TCT GTG TCC T | 153 | 60 | intron seperated<br>exon1-exon2 | 94.12 | 0.999 | 8 logs |
| 69 | Pcna | NM_022381 | rn_pcna_fw<br>rn_pcna_rev | GGG GTG AAG TTT TCT GCG AG<br>GAC AGT GGA GTG GCT TTT GTG | 170 | 60 | intron seperated<br>exon4-exon5 | 98.33 | 0.999 | 7 logs |
| 70 | Pdia3 | NM_017319 | rn_pdia3_fw<br>rn_pdia3_rev | TGC TAG TCG AGT TCT TCG CC<br>TAA GCA CCC GCT TCT TCA CC | 202 | 60 | intron seperated<br>exon1-exon3 | 110.10 | 0.999 | 8 logs |
| 71 | Per1 | NM_001034125.1 | rn_per1_fw<br>rn_per1_rev | ATC CTG AGG ACC GAC CTC TC<br>GAC GTC CTC ATT CAG GGG TG | 218 | 60 | intron seperated<br>exon9-exon11 | 91.00 | 0.999 | 7 logs |
| 72 | Per3 | NM_023978.2 | rn_per3_fw<br>rn_per3_rev | AAG CCA AAC CCA GAG GCA G<br>CGC TCC GGC AAC ACT TT | 219 | 60 | intron seperated<br>exon14-exon16 | 92.55 | 0.999 | 7 logs |
| 73 | Pms1 | NM_001009535 | rn_pms1_fw<br>rn_pms1_rev | ACA TGG AGT CTG TTG AGC GG<br>ATG GGG TAC AAG CGG GTA GA | 220 | 60 | intron seperated<br>exon6-exon8 | 96.48 | 0.993 | 8 logs |
| 74 | Pms2 | NM_001105908 | rn_pms2_fw<br>rn_pms2_rev | CCA GAA GCA GCA TTT CAG GC<br>TCC ACA CAT TCC GAG TCC AC | 205 | 60 | intron seperated<br>exon6-exon9 | 91.34 | 0.998 | 7 logs |
| 75 | Pold3 | NM_001024750 | rn_pold3_fw<br>rn_pold3_rev | TGA GAC ACA AGC CAG TGA GC<br>TAC TCA TGG CGG CTT TTC CA | 242 | 60 | intron seperated<br>exon6-exon7 | 90.32 | 0.998 | 7 logs |
| 76 | Pole | NM_001107152 | rn_pole_fw<br>rn_pole_rev | GCC TCT TGC CAG AGC TAC TT<br>AGT GAT CAT TCC AGG AAG GGC | 180 | 60 | intron seperated<br>exon43-exon45 | 85.51 | 0.998 | 7 logs |
| 77 | Polh | NM_001108204 | rn_polh_fw<br>rn_polh_rev | CTT CTG CTG GCA CAA GTT CG<br>TTC CTG CAC AGC ATT GGT CA | 159 | 60 | intron seperated<br>exon3-exon4 | 82.90 | 0.997 | 7 logs |
| 78 | PolI | NM_001106137 | rn_poli_fw<br>rn_poli_rev | CGA ATC GCT TCG TCC AGA GT<br>GGA CGA GCT GAG GAC ACT TT | 211 | 60 | intron seperated<br>exon2-exon3 | 95.13 | 0.994 | 7 logs |
| 79 | Prkdc | NM_001108327 | rn_prkdc_fw<br>rn_prkdc_rev | ACT CCG AAA GAA CTG GAA GGA<br>ATC CAG CCA ATG AAC TGC CA | 194 | 60 | intron seperated<br>exon71-exon73 | 91.86 | 0.992 | 8 logs |
| 80 | Rplp1 | NM_001007604 | rn_rplp1_fw<br>rn_rplp1_rev | CCT TCC GAG GAA GCT AAG GC<br>AAG CCA GGC CAG AAA GGT TC | 217 | 60 | intron seperated<br>exon1-exon2 | 96.39 | 0.992 | 8 logs |
| 81 | Sec62 | NM_001034129 | rn_sec62_fw<br>rn_sec62_rev | TGC AGT AAT AGC TGC CAC CC<br>AGG AGT CAA TGA AGC CCA CG | 218 | 60 | intron seperated<br>exon11-exon12 | 107.32 | 0.998 | 8 logs |
| 82 | Sec63 | NM_001107637 | rn_sec63_fw<br>rn_sec63_rev | AAG GAC GAT GGC AGT GAC AG<br>GTG CAG GGA ACT TCA GCT CT | 193 | 60 | intron seperated<br>exon17-exon19 | 107.56 | 0.999 | 8 logs |
| 83 | Serp1 | NM_030835 | rn_serp1_fw<br>rn_serp1_rev | CGA GAA ATG CCC CCG AAG AA<br>CCA GGG CAT CGA ACA TCA GG | 194 | 60 | intron seperated<br>exon2-exon3 | 105.48 | 0.997 | 8 logs |
| 84 | Sirt1 | NM_001107627 | rn_sirt1_fw<br>rn_sirt1_rev | TAT GCT CGC CTT GCT GTG GA<br>CAC TGC ACA GGC ACA TAC TGG | 213 | 60 | intron seperated<br>exon3-exon4 | 95.39 | 0.999 | 8 logs |
| 85 | Tnf | NM_012675 | rn_tnf_fw<br>rn_tnf_rev | ACG TCG TAG CAA ACC ACC AA<br>AAA TGG CAA ATC GGC TGA CG | 210 | 60 | intron seperated<br>exon3-exon4 | 94.36 | 0.999 | 8 logs |
| 86 | Tp53 | NM_030989 | rn_tp53_fw<br>rn_tp53_rev | GCT CCC CTG AAG ACT GGA TAA<br>TCC TCT GGG CCT TCT AAC AAC | 208 | 60 | intron seperated<br>exon1-exon4 | 111.89 | 0.997 | 8 logs |

| | | | | | | | | | | |
|---|---|---|---|---|---|---|---|---|---|---|
| 87 | Tp53bp2 | XM_223012 | rn_tp53bp2_fw | GAG TCA GGA GCA GCA GTG TT | 218 | 60 | intron seperated | 100.79 | 0.996 | 8 logs |
| | | | rn_tp53bp2_rev | GCC ATG CAG TCT CCT TCC TT | | | exon16-exon17 | | | |
| 88 | Tradd | NM_001100480 | rn_tradd_fw | TAC CTA GCT GAG CTG CTG GA | 202 | 60 | | 103.33 | 0.998 | 8 logs |
| | | | rn_tradd_rev | TGA GAA TGA GCC AAG GGC AG | | | exon4 | | | |
| 89 | Ube2g2 | NM_001106380 | rn_ube2g2_fw | CCC TTT GAG CCC TCC GAA AA | 181 | 60 | intron seperated | 107.86 | 0.994 | 5 logs |
| | | | rn_ube2g2_rev | CAC CAC CGA AAG CAG GAT CT | | | exon4-exon5 | | | |
| 90 | Ube2j2 | NM_001007655 | rn_ube2j2_fw | AGG CCC TGA GAT GAC TCC TT | 197 | 60 | intron seperated | 110.26 | 0.999 | 8 logs |
| | | | rn_ube2j2_rev | GAA TGG TGG AGA CGG ACC AC | | | exon4-exon6 | | | |
| 91 | Ubxn4 | NM_001012025 | rn_ubxn4_fw | TGG CTG CAA GTT GGG AAG AT | 193 | 60 | intron seperated | 104.16 | 0.998 | 8 logs |
| | | | rn_ubxn4_rev | TCT GCA GAA ACA CTC CCT GC | | | exon2-exon4 | | | |
| 92 | Ufd1l | NM_053418 | rn_ufd1l_fw | GTC AGA TGT GGA GAA AGG AGG G | 183 | 60 | intron seperated | 97.32 | 0.999 | 8 logs |
| | | | rn_ufd1l_rev | ATC CAG TGG GGG AGG TAA CA | | | exon2-exon4 | | | |
| 93 | Xbp1 | NM_001004210 | rn_xbp1_fw | GCA GCA AGT GGT GGA TTT GG | 219 | 60 | intron seperated | 101.98 | 0.997 | 8 logs |
| | | | rn_xbp1_rev | CGT AGT CTG AGT GCT GCG G | | | exon2-exon4 | | | |
