# Supplementary material for: Transcriptomic Analysis of Intestinal Tissues from Two 90-Day Feeding Studies in Rats Using Genetically Modified MON810 Maize Varieties
Source: Front Genet. 2017 Dec 19;8:222. doi: 10.3389/fgene.2017.00222 (PMC5742243; doi:10.3389/fgene.2017.00222)
Supplement: Supplementary file 2 [file Data_Sheet_1.DOCX]

**Electronic Supplementary Data 1**

**Transcriptomic analysis of intestinal tissues from two 90-day feeding studies in rats using genetically modified MON810 maize varieties**

Jutta Sharbati*^1^, Marc Bohmer*^1^, Nils Bohmer^1^, Andreas Keller^2^, Christina Backes^2^, Andre Franke^3^, Pablo Steinberg^4^, Dagmar Zeljenková^5^, Ralf Einspanier^1^

^1^ Institute of Veterinary Biochemistry, Freie Universitaet Berlin, Berlin, Germany

^2^ Chair for Clinical Bioinformatics, Saarland University, Saarbruecken, Germany

^3^ Institute of Clinical Molecular Biology, Christian-Albrechts-University of Kiel, Kiel, Germany

^4^Institute for Food Toxicology & Analytical Chemistry, University of Veterinary Medicine Hannover, Hannover, Germany

^5^ Faculty of Public Health, Slovak Medical University in Bratislava, Bratislava, Slovakia

*These authors contributed equally to this work

Corresponding author(s):

Ralf Einspanier

ralf.einspanier@fu-berlin.de

Institute of Veterinary Biochemistry

Tel. 00493083862575

Fax. 00493083862584


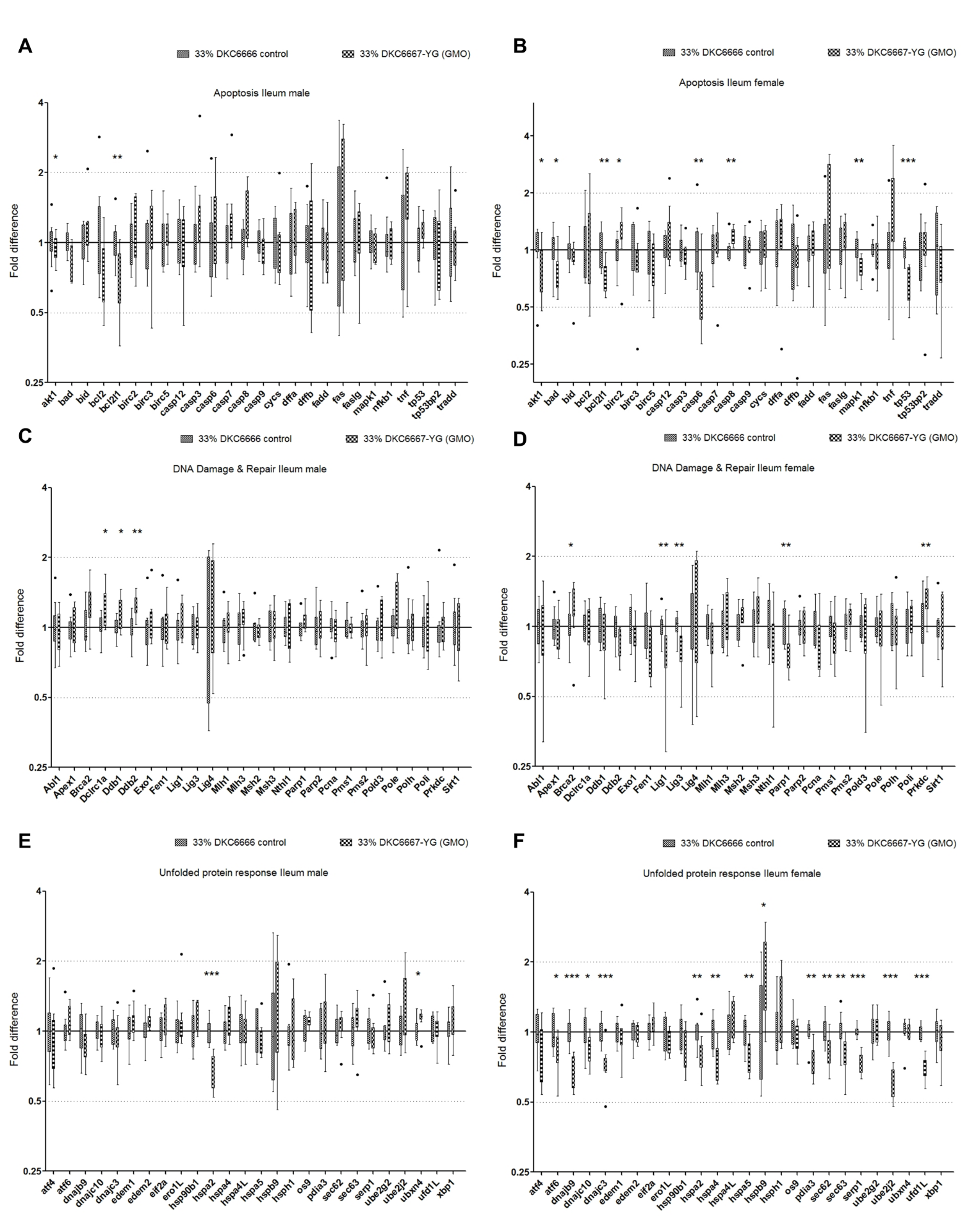


**Akt1**

**Bad**

**Bid**

**Bcl2**

**Bcl2l1**

**Birc2**

**Birc3**

**Birc5**

**Casp12**

**Casp3**

**Casp6**

**Casp7**

**Casp8**

**Casp9**

**Cycs**

**Dffa**

**Dffb**

**Fadd**

**Fas**

**Faslg**

**Mapk1**

**Nfkb1**

**Tnf**

**Tp53**

**Tp53bp2**

**Tradd**

**Akt1**

**Bad**

**Bid**

**Bcl2**

**Bcl2l1**

**Birc2**

**Birc3**

**Birc5**

**Casp12**

**Casp3**

**Casp6**

**Casp7**

**Casp8**

**Casp9**

**Cycs**

**Dffa**

**Dffb**

**Fadd**

**Fas**

**Faslg**

**Mapk1**

**Nfkb1**

**Tnf**

**Tp53**

**Tp53bp2**

**Tradd**

**Atf4**

**Atf6**

**Dnajb9**

**Dnajc10**

**Dnajc3**

**Edem1**

**Edem2**

**Eif2a**

**Ero1L**

**Hsp90b1**

**Hspa2**

**Hspa4**

**Hspa4L**

**Hspa5**

**Hspb9**

**Hsph1**

**Os9**

**Pdia3**

**Sec62**

**Sec63**

**Serp1**

**Ube2g2**

**Ube2j2**

**Ubxn4**

**Ufd1L**

**Xbp1**

**Atf4**

**Atf6**

**Dnajb9**

**Dnajc10**

**Dnajc3**

**Edem1**

**Edem2**

**Eif2a**

**Ero1L**

**Hsp90b1**

**Hspa2**

**Hspa4**

**Hspa4L**

**Hspa5**

**Hspb9**

**Hsph1**

**Os9**

**Pdia3**

**Sec62**

**Sec63**

**Serp1**

**Ube2g2**

**Ube2j2**

**Ubxn4**

**Ufd1L**

**Xbp1**

**Supplementary Fig. S1**: Individual boxplot analysis of targeted pathway RT-qPCR arrays in ileum. A: apoptosis male; B: apoptosis female; C: DNA Damage and Repair male; D: DNA Damage and Repair female; E: unfolded Protein Response male; F: unfolded protein response female. Asterisks indicate statistical significance between samples (*: P <0.05; **: P <0.01; ***: P <0.001, Mann-Whitney U test).


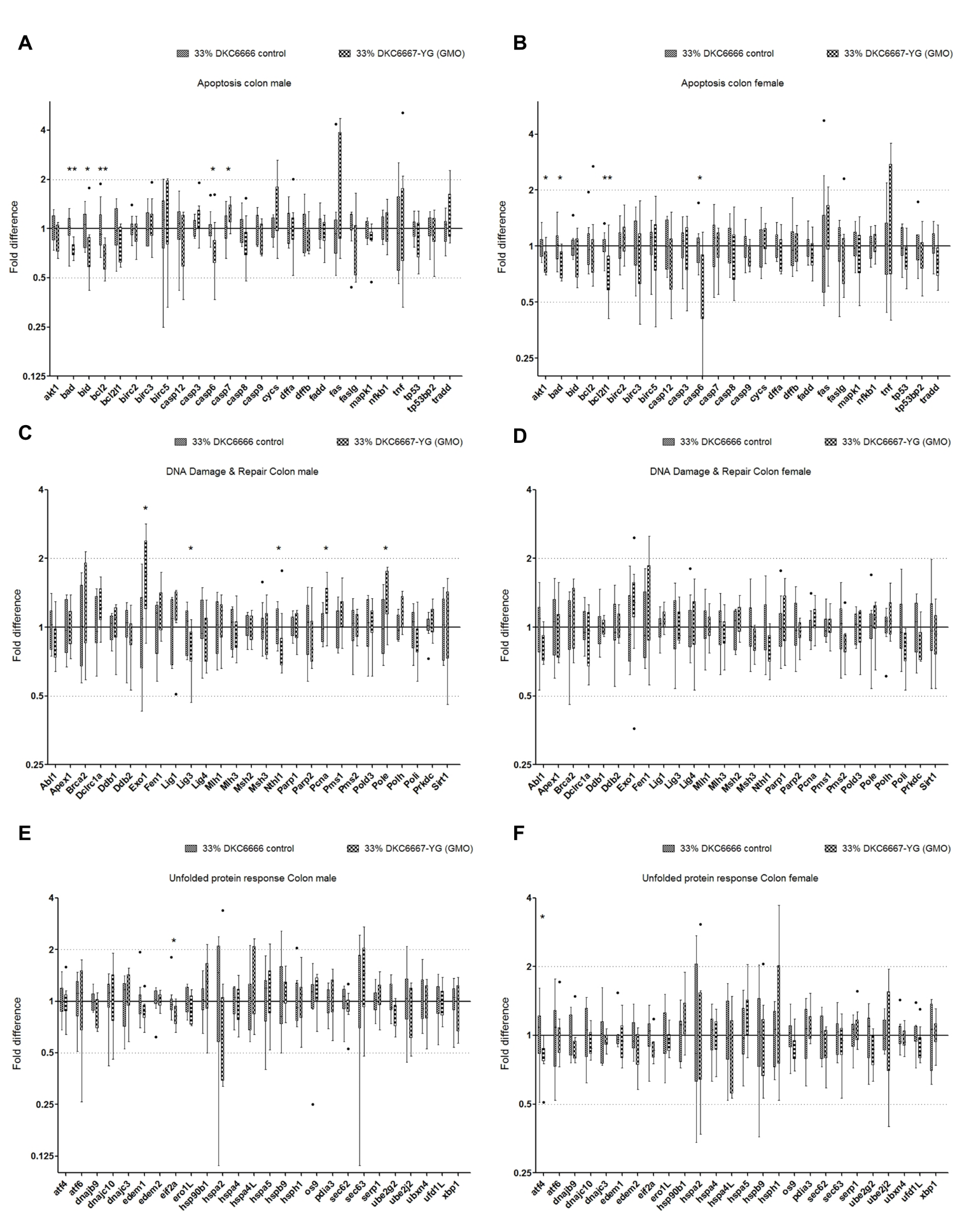


**Akt1**

**Bad**

**Bid**

**Bcl2**

**Bcl2l1**

**Birc2**

**Birc3**

**Birc5**

**Casp12**

**Casp3**

**Casp6**

**Casp7**

**Casp8**

**Casp9**

**Cycs**

**Dffa**

**Dffb**

**Fadd**

**Fas**

**Faslg**

**Mapk1**

**Nfkb1**

**Tnf**

**Tp53**

**Tp53bp2**

**Tradd**

**Akt1**

**Bad**

**Bid**

**Bcl2**

**Bcl2l1**

**Birc2**

**Birc3**

**Birc5**

**Casp12**

**Casp3**

**Casp6**

**Casp7**

**Casp8**

**Casp9**

**Cycs**

**Dffa**

**Dffb**

**Fadd**

**Fas**

**Faslg**

**Mapk1**

**Nfkb1**

**Tnf**

**Tp53**

**Tp53bp2**

**Tradd**

**Atf4**

**Atf6**

**Dnajb9**

**Dnajc10**

**Dnajc3**

**Edem1**

**Edem2**

**Eif2a**

**Ero1L**

**Hsp90b1**

**Hspa2**

**Hspa4**

**Hspa4L**

**Hspa5**

**Hspb9**

**Hsph1**

**Os9**

**Pdia3**

**Sec62**

**Sec63**

**Serp1**

**Ube2g2**

**Ube2j2**

**Ubxn4**

**Ufd1L**

**Xbp1**

**Atf4**

**Atf6**

**Dnajb9**

**Dnajc10**

**Dnajc3**

**Edem1**

**Edem2**

**Eif2a**

**Ero1L**

**Hsp90b1**

**Hspa2**

**Hspa4**

**Hspa4L**

**Hspa5**

**Hspb9**

**Hsph1**

**Os9**

**Pdia3**

**Sec62**

**Sec63**

**Serp1**

**Ube2g2**

**Ube2j2**

**Ubxn4**

**Ufd1L**

**Xbp1**

**Supplementary Figure S2:** Individual boxplot analysis of targeted pathway RT-qPCR arrays in colon. A: apoptosis male; B: apoptosis female; C: DNA Damage and Repair male; D: DNA Damage and Repair female; E: unfolded Protein Response male; F: unfolded protein response female. Asterisks indicate statistical significance between samples (*: P <0.05; **: P <0.01; ***: P <0.001, Mann-Whitney U test).


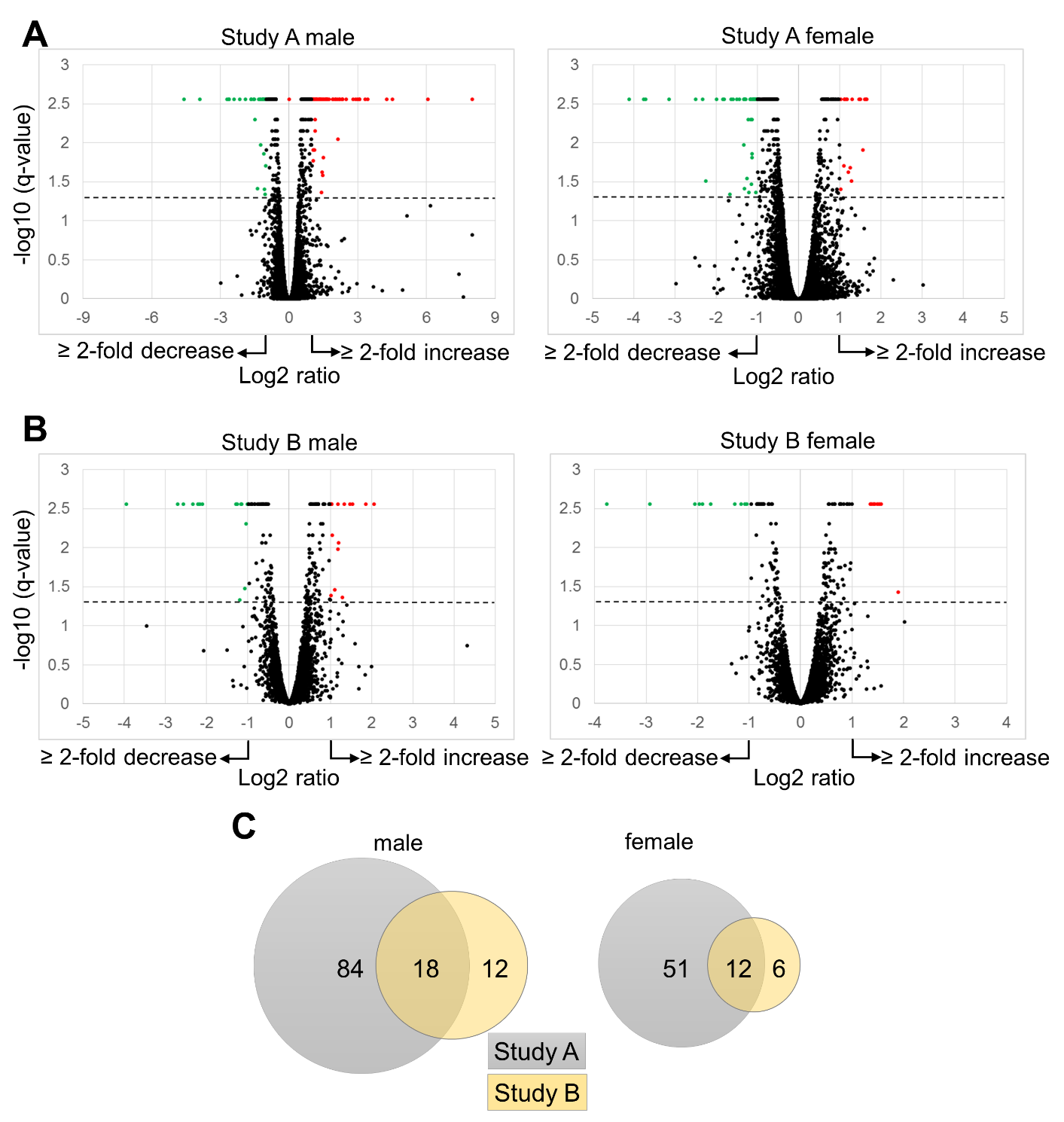


**Supplementary Figure S3:** Fig. 2 Global analysis of ileal transcripts by RNAseq in studies A (a) and B (b). a) Volcano plots demonstrate the distribution of log2 ratios. Green and red dots represent genes with significant down-regulation or up-regulation in GM fed animals, respectively (log2 ratios > 1 or < -1; q ≤ 0.05). b) Comparison of the total number of differentially expressed genes in studies A and B demonstrated by a Venn’s diagram based on q-values ≤ 0.05 and log2 ratios of > 1 or <-1.

**Supplementary Figure S4:** Correlation plot of fold changes in RT-qPCR and RNAseq studies (Spearman's rank correlation coefficient: 0.94). The dots represent the comparison of individual NGS vs. qPCR fold changes for all seven selected factors of the circadian clock (Arntl, Cry1, Npas2, Nr1d1, Per1, Dbp, Per3) resulting in 112 measurements (N= 16 rats).

**
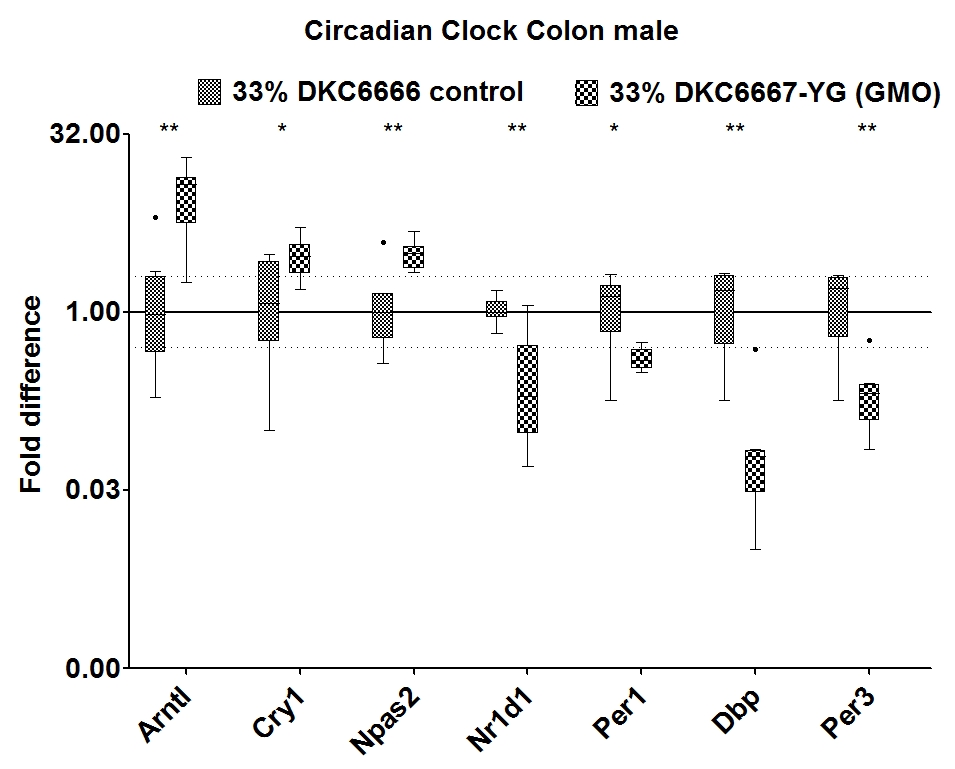

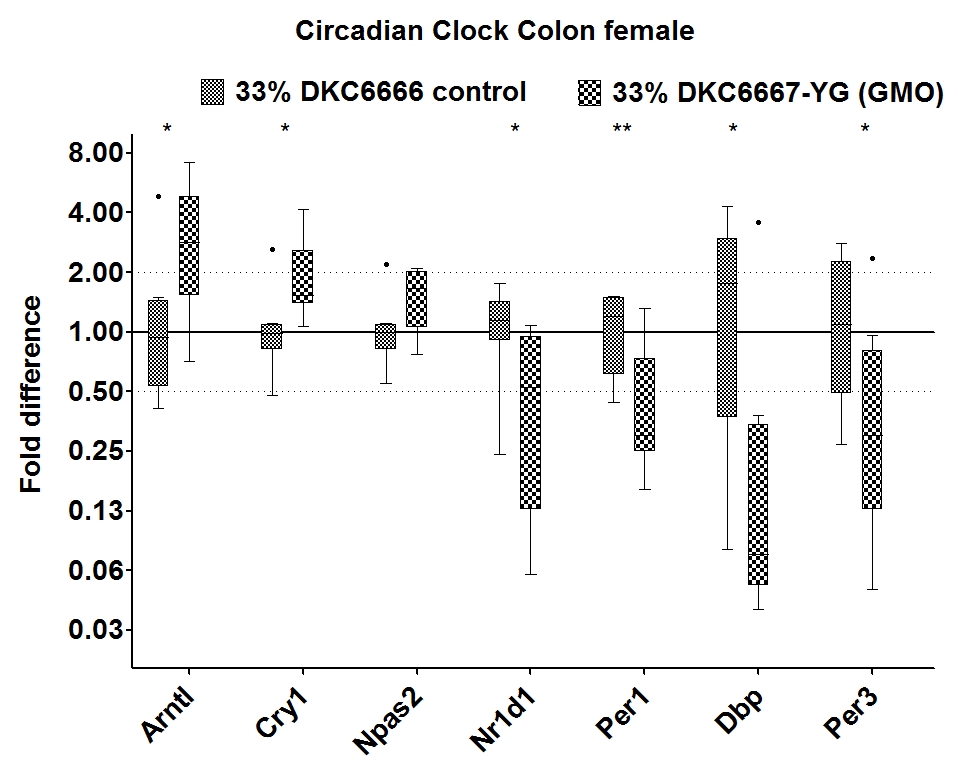
**

**Supplementary Figure S5:** Individual boxplot analysis of the circadian clock pathway RT-qPCR arrays in colon for male (upper panel) and female (lower panel) rats. Asterisks indicate statistical significance between samples (*: P <0.05; **: P <0.01; ***: P <0.001, Mann-Whitney U test).

**Supplementary Figure S6:** A-D: Log2-Fold Changes of selected genes from the TissGDB (cancer type COAD). Gene expression data (RNA-seq) from both studies A and B demonstrate near-complete absence of significant and more than 2-fold differences (log 2 < -1 or log2 > 1) in expression of genes, that are associated with colorectal cancer. The grey line indicates the cutoff for significance (q-values < 0,05). A: An exception is the expression of Cyp2s1 and Agp8 in male animals of study A, both having a significant 2,8- and 4,3-fold higher expression in the GMO-group compared to controls, respectively.
